# In Vitro and Ex Vivo Evaluation of Nepafenac-Based Cyclodextrin Microparticles for Treatment of Eye Inflammation

**DOI:** 10.3390/nano10040709

**Published:** 2020-04-09

**Authors:** Blanca Lorenzo-Veiga, Patricia Diaz-Rodriguez, Carmen Alvarez-Lorenzo, Thorsteinn Loftsson, Hakon Hrafn Sigurdsson

**Affiliations:** 1 Faculty of Pharmaceutical Sciences, University of Iceland, Hofsvallagata 53, IS-107 Reykjavik, Iceland; blv3@hi.is (B.L.-V.); thorstlo@hi.is (T.L.); 2 Departamento de Ingeniería Química y Tecnología Farmacéutica, Facultad de Ciencias de la Salud, Universidad de la Laguna (ULL), Campus de Anchieta, 38200 La Laguna (Tenerife), Spain; pdiarodr@ull.edu.es; 3 Departamento de Farmacología, Farmacia y Tecnología Farmacéutica, R+D Pharma Group (GI-1645), Facultad de Farmacia and Health Research Institute of Santiago de Compostela (IDIS), Universidade de Santiago de Compostela, 15782 Santiago de Compostela, Spain; carmen.alvarez.lorenzo@usc.es

**Keywords:** eye drop, cyclodextrin, nepafenac, HET-CAM, ex vivo permeation studies, ocular inflammation

## Abstract

The aim of this study was to design and evaluate novel cyclodextrin (CD)-based aggregate formulations to efficiently deliver nepafenac topically to the eye structure, to treat inflammation and increase nepafenac levels in the posterior segment, thus attenuating the response of inflammatory mediators. The physicochemical properties of nine aggregate formulations containing nepafenac/γ-CD/hydroxypropyl-β (HPβ)-CD complexes as well as their rheological properties, mucoadhesion, ocular irritancy, corneal and scleral permeability, and anti-inflammatory activity were investigated in detail. The results were compared with a commercially available nepafenac suspension, Nevanac^®^ 3 mg/mL. All formulations showed microparticles, neutral pH, and negative zeta potential (–6 to –27 mV). They were non-irritating and nontoxic and showed high permeation through bovine sclera. Formulations containing carboxymethyl cellulose (CMC) showed greater anti-inflammatory activity, even higher than the commercial formulation, Nevanac^®^ 0.3%. The optimized formulations represent an opportunity for topical instillation of drugs to the posterior segment of the eye.

## 1. Introduction

Inflammation of the eye and surrounding tissues is among the ocular pathologies with the highest incidence in ophthalmology, which, deprived of the appropriate treatment, can lead to visual loss [1]. The main symptoms include eye redness, eye pain, itchiness, blurred vision, swelling, and visual distortions [2]. The most common causes of ocular inflammation at the posterior segment of the eye are related to eye disorders such as glaucoma, macular edema, cataract surgery intervention, scleritis, posterior uveitis, and diabetic retinopathy [3,4]. Other causes that affect the anterior segment of the eye include conjunctivitis, ocular infections, anterior and intermediate uveitis, dry eye syndrome, keratitis, use of contact lenses, and trauma [5]. Although inflammation can be triggered by a variety of etiological causes, the symptoms are similar, as they induce similar immunological response [6,7].

Treating inflammation at the posterior segment classically involves ophthalmic steroids as well as nonsteroidal anti-inflammatory drugs (NSAIDs) [8,9]. The repetitive use of traditional corticosteroids such as fluocinolone, dexamethasone, prednisolone, or fluorometholone can lead to undesirable side effects, such as high intraocular pressure, risk of infection, cataract formation, or macular edema [10]. Ophthalmic NSAIDs approved by the US Food and Drug Administration (FDA) used for the treatment of ocular inflammation and pain include diclofenac 0.1%, ketorolorac 0.6%, and bromfenac 0.09% solutions, and nepafenac 0.1% and 0.3% suspensions [11,12]. The use of topical NSAID formulations can lead to cornea infiltrations, cornea melting, or keratitis [13]. Nepafenac is an NSAID prescribed prophylactically as well as post cataract surgery. It is currently approved for treatment of pain and inflammation after cataract surgery and commercialized as an eye drop suspension, Nevanac^®^, in two doses, 1 mg/mL three times per day and 3 mg/mL once per day [14]. The side effects include increased intraocular pressure, decreased visual acuity, and sticky eyes [15]. Sahu et al. [16] analyzed the effects of three topical NSAIDs (ketorolac 0.4%, nepafenac 0.1%, and bromfenac 0.09%) on inflammation after surgery. Their results showed that nepafenac was significantly more effective that the others at reducing anterior chamber redness. Moreover, Modi et al. [17] demonstrated the convenience of instilling nepafenac 0.3% once a day compared to Nevanac^®^ 0.1% three times a day, as they both showed the same efficacy.

**Figure 1:**
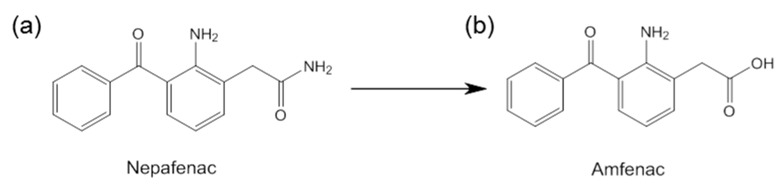
Structures of (**a**) nepafenac and (**b**) amfenac.

Unlike other NSAIDs, nepafenac is a prodrug. After topical ocular treatment, it penetrates the cornea and is transformed by ocular tissue hydrolases into its active metabolite, amfenac (Figure 1), an inhibitor of cyclooxygenase-1 and -2 (COX-1, COX-2) [18,19]. The expression of cyclooxygenase enzyme has been widely studied [19,20]. The activation of COX-1 and COX-2 is involved in prostaglandin production, and therefore in the inflammation process in the eye. Because of eye inflammation, changes to the blood–ocular barrier, ocular angiogenesis, and vascular permeability can occur. Inhibition of COX activity blocks the formation of proinflammatory mediators, including prostaglandins, reducing edema and inflammation [21,22].

Treating the posterior segment in eye diseases by means of topical instillation is still a challenge, due to the different biological membranes and physical boundaries of the eye that restrict drug passage and penetration [23,24,25]. Newly biodegradable nanoparticulated drug systems have been proposed as promising alternatives in the treatment of retinal diseases [26,27,28]. Tahara et al. [29] prepared poly(lactide-co-glycolide) (PLGA) nanoparticles of three corticosteroids (dexamethasone, hydrocortisone acetate and prednisolone acetate) suspended in gels for the treatment of macular edema and studied their ex vivo permeation using rabbit eyes. These polymeric nanoparticles were able to sustain drug delivery to the retina after episcleral administration. Furthermore, Balguri et al. [30] designed chitosan-based solid lipid nanoparticles that were able to deliver indomethacin to the cornea and sclera. Additionally, conventional eye drops are not able to maintain therapeutic concentrations in the ocular tissues due to short contact time and fast elimination [31,32,33]. To overcome these problems, cyclodextrin (CD) nanoparticles have been proposed as one of the best options for topical eye drop instillation of both small-molecule drugs and biomolecules to the eye [34]. An aqueous-based eye drop formulation of 0.2% (w/v) cyclosporine A with 12.5% (w/v) alpha-cyclodextrin (α-CD), various amounts of gamma-cyclodextrin (γ-CD), and 1.4% (w/v) hydrolyzed poly(vinyl alcohol) (PVA) was shown to be well tolerated in rabbits [35,36]. Furthermore, some CD-based eye drop formulations are already commercialized (Clorocil^®^, Voltaren^®^, Vitaseptol^®^, and Indocid^®^) [37]. Combinations of polymers and cyclodextrins have been reported as a strategy to enhance drug permeation in ocular tissues [38,39,40]. Recently, we found that the addition of one or more polymers to nepafenac/γ-CD/hydroxypropyl-β (HPβ)-CD complexes (Table 1) leads to the enhancement of its solubility in water, offering an alternative to current nepafenac eye drops [41], and therefore their efficacy should be further evaluated. The polymer compositions of preliminary eye drop suspensions are summarized in Table 1.

This study was aimed at the development and evaluation of novel CD-based aqueous eye drop formulations containing mucoadhesive polymers (sodium hyaluronate and sodium alginate) and comparing their effectiveness with previous formulations developed to efficiently deliver nepafenac to the eye in order to treat inflammation, increase drug concentration in the posterior segment, and reduce the expression of inflammatory mediators. All aggregate formulations were evaluated for in vitro diffusion studies, rheological and mucoadhesive properties, in vitro anti-inflammatory activity, and ex vivo corneal and scleral permeability studies. They were compared with a commercial Nevanac^®^ 3 mg/mL suspension. To the best of our knowledge, this is the first time that formulations containing γ-CD/HPβ-CD nanoparticles have been evaluated in vitro and ex vivo.

## 2. Materials and Methods

### 2.1. Materials

Nepafenac (98% purity, MW 254.28 g/mol) was acquired from Fagron (Rotterdam, Netherlands); γ-cyclodextrin (γ-CD) was provided by Wacker Chemie (Munich, Germany); 2-hydroxypropyl-β-cyclodextrin, DS 0.62 (HPβ-CD; MW 1380 Da) was kindly donated by Janssen Pharmaceutica (Beerse, Belgium). Methyl cellulose (MC; MW 14,000 Da; viscosity ~15 cPs) was from ICN Biomedicals Inc. (Solon, OH, USA); sodium alginate (SA; MW 80,000–12,000 Da) was from Fagron Iberica (Zaragoza, Spain); sodium hyaluronate (HA; MW 360,000 Da, glucuronic acid 47.4%) was from Guinama (La Pobla de Valbona, Spain).

Benzalkonium chloride (BAK), ethylenediamine-tetraacetic acid disodium salt dihydrate (EDTA), reagent-grade tyloxapol (MW 280.4 g/mol), 87%–90% hydrolyzed poly(vinyl alcohol) (PVA) (average MW 30,000–70,000 Da), hydroxypropyl methylcellulose (HPMC; MW 26,000 Da, viscosity ~100 cPs), polyvinylpyrrolidone (PVP; average MW 40,000 Da), and carboxymethylcellulose (CMC) sodium salt (MW 90,000 Da; low viscosity) were purchased from Sigma-Aldrich (St. Louis, MO, USA). Membrane filters (0.45 µm) were obtained from Phenomenex (Cheshire, UK). Water was purified using reverse osmosis (resistivity > 18 MΩcm; Milli-Q, Millipore^®^, Madrid, Spain). All other reagents were analytical grade.

Carbonate buffer, pH 7.2, was prepared by mixing buffer solution A (100 mL; 1.24 g NaCl, 0.071 g KCl, 0.02 g NaH_2_PO_4_, 0.49 g NaHCO_3_) and buffer solution B (100 mL; 0.023 g CaC_l2_, 0.031 g MgC_l2_).

BALB/3T3 clone A31 mouse fibroblasts (ATCC CCL-163™) and THP-1 monocytes (ATCC TIB-202™) were purchased from the American Type Culture Collection (ATCC, Manassas, VA, USA). Fetal bovine serum (FBS), antibiotic solution (penicillin 10,000 units/mL and streptomycin 10.00 μg/mL), lipopolysaccharides (LPS) from E. coli, phorbol 12-myristate 13-acetate (PMA), tris hydrochloride, and lauryl sulfate sodium salt (SDS) were acquired from Sigma-Aldrich (St. Louis, MO, USA). Dulbecco’s Modified Eagle’s Medium with Ham’s F-12 Nutrient Mixture (DMEM/F12) and RPMI 1640 were supplied by Gibco (Thermo Fisher, Paisley, UK). WST-1 Cell Proliferation Reagent was purchased from La Roche (Manheim, Germany).

### 2.2. Nepafenac Eye Drop Preparation

Nepafenac (18 mg) was added to 6 mL of an aqueous 15%γ-CD/8%HP-βCD (w/v) solution containing different polymers (Table 2), 0.1% (w/v) EDTA, 0.02% (w/v) BAK, and 0.04% (w/v) NaCl.

Subsequently, suspensions were placed in an ultrasonic water bath (Branson 3510 Ultrasonic Cleaner, Marshall Scientific, Hampton, NH, USA) at 60 °C for 60 min. They were cooled down to room temperature and kept in a shaker (Unitronic, JP Selecta, Spain) under constant agitation for 7 days at 37 °C. After this, suspensions were filtered (Acrodisc^®^ Syringe Filter, 0.22 µm; GHP Minispike, Waters) and centrifuged at 4000 rpm for 15 min at 25 °C (centrifuge model 5804R, Eppendorf AG, Germany), and supernatant was diluted with Milli-Q water. The apparent nepafenac solubility was determined by UV-Vis spectroscopy at 254 nm using a standard calibration curve previously validated in triplicate in the range 3–25 µg/mL.

### 2.3. Physicochemical Characterization

#### 2.3.1. Particle Size Analysis

Particle size and size distribution of formulations A1 to A9 and Nevanac was measured by dynamic light scattering (DLS) using a Nanotrac Wave particle analyzer (Microtrac, York, PA, USA). Samples that were previously filtered were diluted with Milli-Q water, and measurements were carried out at 25 °C with a 780 nm laser and 180° scattering angle. Each measurement was done in triplicate.

#### 2.3.2. Zeta Potential and pH

Zeta potential of formulations A1 to A9 was recorded using a Zetasizer^®^ 3000HS. pH was measured with a GLP22 pH meter (Crison Instruments, Barcelona, Spain). All measurements were done in triplicate at 25 °C.

#### 2.3.3. Rheological Analysis

Rheological characterization of formulations was carried out using a Rheolyst AR-1000N rheometer (TA Instruments, Newcastle, UK) equipped with an AR2500 data analyzer, a Peltier plate, and a cone (6 cm diameter, 2.1°). First, storage (G′) and loss (G″) moduli were recorded at 37 °C and 0.1 Pa applying angular frequency sweeps from 0.1 to 50 rad/s. Viscosity and flow curves were performed under rotational runs at 37 °C for 2 min with shear stress in the range 0.1 to 200 s^–1^. Data analysis was carried out using Rheology Advantage data analysis software. Experiments were performed using 1.5 mL for each formulation.

#### 2.3.4. In Vitro Mucoadhesive Studies

Mucoadhesion strength was evaluated in triplicate using a TA.XT Plus Texture analyzer (Stable Micro Systems Products, Godalming, UK) following methods previously described by Akhter et al. [42] and Campaña-Seoane et al. [43] with some modifications. Bovine corneas were placed beneath double-sided tape at the end of the probe. To simulate the eye drop application, 15 µL of each formulation was placed at the bottom of a Petri dish. Mucoadhesion strength was determined as the detachment force needed to separate the formulation from the cornea after applying a force of 0.5 N for 60 seconds.

### 2.4. Ocular Tolerance Test (HET-CAM assay)

The ocular irritation test was carried out as previously reported [44]. Briefly, 200 µL of each formulation was tested, at least in duplicate, on chorioallantoic membranes (Hen’s Egg Test-Chorioallantoic Membrane, HET-CAM) of chicken eggs after 10 days of incubation at 37 °C and 60% RH. The time and severity of injuries after the addition of each formulation was recorded. The irritation score (IS) was calculated as follows (34):

(1) $$\mathrm{IS}=\frac{(301\;-\;\mathrm{tH})\;\times \;5}{300}+\frac{\left(301\;-\;\mathrm{tL}\right)\;\times \;7}{300}+\frac{\left(301\;-\;\mathrm{tC}\right)\;\times \;9}{300}$$

where tH, tL, and tC are the time (in seconds) needed for the appearance of hemolysis, lysis, and coagulation, respectively. Depending on IS values, formulations were classified as non-irritating (IS < 1), mildly irritating (1 ≤ IS < 5), moderately irritating (5 ≤ IS < 10), or severely irritating (IS > 10).

### 2.5. In Vitro Cell Viability

The cytocompatibility of cyclodextrin formulations was evaluated on BALB/3T3 clone A31 (ATCC^®^ CCL-163TM) murine fibroblasts using the WST-1 test. BALB 3T3 cells were cultured in DMEM/F12 culture medium (Corning) supplemented with 10% fetal bovine serum (Hyclone) and 1% penicillin/streptomycin (Gibco). They were seeded in a 96-well plate at 1.5 × 10^4^ cells/well. To allow complete cell attachment, cells were incubated 4 h at 37 °C and 5% CO_2_. Aliquots of A1 to A9 formulations, Nevanac 3 mg/mL suspension, and control (DMEM/F12) were diluted 1:50, 1:100, and 1:150 times, respectively, with complete cell culture medium to be below the IC_50_ of nepafenac, to ensure that nepafenac was not in cytotoxic concentrations, and added to cell monolayers [45]. DMEM/F12 medium was used as control. After 24 hours of incubation with the formulations, WST-1 reagent (Roche) was added and the assay was carried out according to the manufacturer’s instructions. The absorbance was measured at 450 nm using a Model 680 microplate reader from Bio-Rad (Hercules, CA, USA) and Microplate Manager software (Version 5.2.1, BioRad, CA, USA).

### 2.6. Diffusion Assays

Nepafenac diffusion tests from eye drop formulations were performed in triplicate in vertical Franz diffusion cells fitted with cellulose acetate membrane filters (0.45 µm pore size, 25 mm diameter). Membrane filters were soaked in the receptor medium for 1 hour before starting the experiment. The donor phase consisted of aliquots of 1.00 mL of the test formulation. The receptor phase was 6.00 mL of 2.5% (w/v) γ-CD/HPβ-CD ratio (80/20) aqueous medium to ensure sink conditions, and kept at 37 °C and under magnetic stirring at 300 rpm. The diffusion area was 0.786 cm^2^. Samples (1 mL) were taken from the receptor phase at 30, 60, 90, 120, 180, 210, 240, 300, and 360 min and replaced with fresh medium. Commercial eye drops, Nevanac 3 mg/mL, were also tested. Nepafenac content was determined by UV-VIS spectrophotometry at 254 nm using a method previously validated with standard solutions in the range of 3–25 µg/mL.

Diffusion coefficients (D) were estimated from the Higuchi equation:

(2) $$\frac{Q}{A}{=2C}_{0}{\left(\frac{\mathrm{Dt}}{\pi }\right)}^{\frac{1}{2}}$$

where Q is the amount of nepafenac (g) released by time t (min), A is the diffusion area (cm^2^), C_0_ is the initial concentration of nepafenac in the formulation (g/mL), and D is the diffusion coefficient (cm^2^/min).

### 2.7. Ex Vivo Corneal and Scleral Permeability

Ex vivo corneal and scleral permeability studies of selected nepafenac formulations were carried out using fresh bovine eyes from a local slaughterhouse. The eyes were kept in phosphate-buffered saline (PBS) solution with antibiotics previously added (penicillin 100 IU/mL and streptomycin 100 μg/mL) and maintained in an ice bath during transport. Corneas and scleras were isolated, washed with PBS, and placed on vertical diffusion Franz cells. Both receptor and donor phases were filled with carbonate buffer, pH 7.2, following the bovine corneal opacity and permeability (BCOP) protocol, placed in a bath at 37 °C, and kept under magnetic stirring for 1 h in order to balance ocular tissues. After this, the buffer in the donor chamber was completely removed and replaced by the formulations (2 mL). Chambers were covered with parafilm to prevent evaporation (0.785 cm^2^ area available for permeation). Samples (1 mL) were removed from the receptor chamber at 0.5, 1, 2, 3, 4, 5, and 6 h, replacing the same volume with carbonate buffer each time, and taking care to remove bubbles from the diffusion cells. All experiments were carried out in triplicate.

Permeated nepafenac was quantified at 254 nm using a Jasco HPLC system (AS-4140 autosampler, PU-4180 pump, LC-NetII/ADC interface box, CO-4060 column oven, MD-4010 photodiode array detector), with a C18 column (Waters Symmetry C18, 5 μm, 3.9 × 150 mm) and ChromNAV software. The mobile phase consisted of acetonitrile: water (50:50) at a flow rate of 1 mL/min and 90 µL for injection volume and retention time of 1.9 min.

After a 6 h permeation test, aliquots of the donor chambers were taken for HPLC analysis. Previously injected corneas and scleras were excised and nepafenac content was extracted in tubes with 3 mL of ethanol/water (50:50 v/v) mixture for 24 h at 37 °C, and sonication was applied for 90 min in an ultrasound bath at 37 °C. Afterwards, tubes were centrifuged (1000 rpm, 5 min, 25 °C), and the supernatant was filtered (Acrodisc^®^ syringe filter, 0.22 µm GHP Minispike, Waters) into small Eppendorfs, centrifuged again (14,000 rpm, 20 min, 25 °C), and filtered to be measured by HPLC.

The apparent permeability coefficient (P_app_) was calculated from the flux (J) according to Equation (3):

(3) $$P_{\mathrm{app}}=\frac{J}{C_{0}}$$

where J is the flux, calculated as the slope (Q/t) of the linear section of the amount of drug in the receptor chamber (Q) versus time (t), and C_0_ is the initial concentration of nepafenac in the donor phase. Each experiment was performed in triplicate and the results are reported as mean values ± standard deviation (SD).

### 2.8. Human Monocytes

#### 2.8.1. Differentiation into Macrophages

THP-1 human monocytes (ATCC TIB-202™) were cultured in RPMI 1640 (Gibco) supplemented with 10% fetal bovine serum (Hyclone), 2-mercaptoethanol (0.05 mM; Gibco), and 1% penicillin-streptomycin (Gibco). Phorbol 12-myristate 13-acetate (PMA; Sigma-Aldrich) 200 nM was used to promote the differentiation of THP-1 monocytes into macrophages [46]. Previously, monocytes had been counted in a Coulter Multisizer3 (Beckman Coulter, Indianapolis, IN, USA) and cell density was adjusted to 200,000 cells per mL. Then, PMA 200 nM was added to differentiate THP-1 cells into macrophages and they were incubated for 72 h at 37 °C.

#### 2.8.2. Anti-inflammatory Activity

After macrophage differentiation, PMA solution was removed and cell monolayers were washed with Dulbecco’s phosphate buffered saline (DPBS) and trypsinized following standard protocols. Cells were seeded into 48-well plates at 4.5 × 10^4^ cells/well. To induce an inflammatory response, macrophages were treated with 100 ng/mL of lipopolysaccharides (LPS) from Escherichia coli O111:B4 (St. Louis, MO, USA, Sigma-Aldrich) and incubated at the same time for 24 h at 37 °C and 5% CO_2_ with the samples. Cells treated with only LPS served as positive controls, while unstimulated cells (without LPS) were used as negative controls. Formulations A2, A3, A5, A8, and A9 were selected for anti-inflammatory efficacy, and their corresponding blank formulations (without the drug) were also tested as controls.

After incubation, cell culture supernatants were collected and stored at –150 °C until cytokine assessment. The secretion of 3 inflammatory mediators, PEG-2, IL-6, and IL-1ra, was analyzed. The concentration of PEG-2 was studied using an EIA assay (Arbor Assays), while IL-6 and IL-1ra were analyzed by specific ELISAs (Sigma, St. Louis, MO, USA) after adequate dilution following the manufacturer’s instructions.

### 2.9. Statistical Analysis

Data are presented as mean ± standard deviation (SD). The effect of formulation composition on anti-inflammatory response was analyzed using ANOVA and multiple range test (Statgraphics Centurion XVI 1.16.1.11, StatPoint Technologies Inc., Warrenton, VA, USA). Differences were considered significant at *p* < 0.05.

## 3. Results and Discussion

### 3.1. Solubility of Nepafenac Eye Drops and Their Characterization

The apparent solubility, zeta potential, and pH of the designed formulations of nepafenac are summarized in Table 3.

In the formation of ternary complexes, drug-CD-polymer has been widely explored to enhance solubility and dissolution of poorly soluble drugs [47,48]. One aim of this study was to elucidate if the addition of hydrophilic polymers to nepafenac/CD complex could enhance its solubility as well as increase the residence time at the ocular tissues. For that, different polymers and concentrations were tested. As shown in Table 3, the apparent solubility of nepafenac was increased in all formulations compared to aqueous solubility, which has been reported to be 0.0197 mg/mL at 25 °C in water [49]. The addition of SA displayed the lowest solubility enhancement (formulation A7, 1.68 ± 0.01 mg/mL) while the combination of SA with CM or CMC and HA led to higher complex solubility (1.89 ± 0.05 and 2.61 ± 0.02 mg/mL, respectively). These differences are due to the increase of cyclodextrin complexing-power for nepafenac. Regarding formulations containing CMC, formulation A9 (CMC, PVA, and MC), A5 (CMC, PVP, and HPMC), and A3 (CMC, PVA, and tyloxapol) exhibited the highest solubility, 2.61, 2.50, and 2.23 mg/mL, respectively. These results support our previous preliminary studies, which showed that formulations containing CMC and/or PVA led to the highest solubilization of nepafenac.

All formulations showed negative zeta potential, in good agreement with the anionic or nonionic nature of the polymers and surfactants involved. Formulation A6 showed the highest absolute value of zeta potential, –27.4 ± 1.7 mV. Although a high absolute value of zeta potential is related to the high stability of nanoaggregates, other factors such as ionic strength, pH, or amount of encapsulated drug can influence aggregation behavior [50].

Moreover, all pH values were about 6.08–6.21, i.e., in a range adequate for ocular administration [51]. Particle size and size distribution were also evaluated by DLS (Table 4).

All formulations tested presented microparticles (approx.5–6 µm), and formulations A3, A5, and A9, which contained PVA and CMC or PVP and CMC, displayed also small portion of smaller particles (less than 1 µm). Compared to the particle size of Nevanac reported by Shelley et al. [51], the increase in particle size of our formulations could be attributed to the different polymers used. In fact, a similar size range was reported by Jansook and co-workers [52] after the preparation of irbersartan eye drops also containing 15% γ-CD. Moreover, all formulations showed a high polydispersity index, which may be due to the nanoaggregates forming and disrupting continuously.

### 3.2. Rheological Characterization

**Figure 2:**
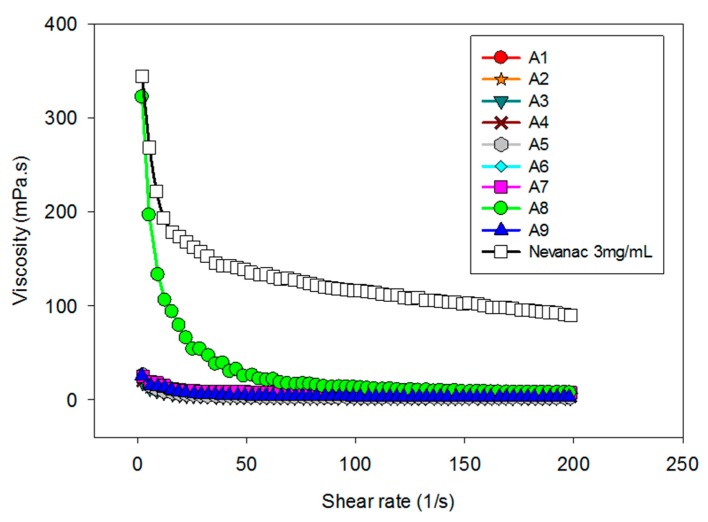
Dependence of viscosity on shear rate conditions of eye drops measured at 37 °C.

Short precorneal residence time limits the ocular bioavailability of drugs formulated as conventional topical eye drops. One strategy to prolong precorneal residence relies on the addition of polymers that can increase viscosity and therefore drug retention in the ocular sac [53]. Viscosity profiles of formulations A1 to A9 and Nevanac are displayed in Figure 2.

It is known that upon application of mechanical force such as eye blinking, viscosity of the natural tear film decreases in a pronounced manner. Eye drops should ideally display pseudoplastic behavior to prevent their removal under blinking conditions [50]. As shown in Figure 2, at 37 °C, all formulations showed pseudoplastic behavior, high viscosity during interblinking (shear rate 0.03 s^–1^), and low viscosity during blinking (shear rate 4250–28,500 s^–1^), which is appropriate for eye drop formulations [51]. Regarding viscosity, Nevanac showed the highest viscosity at 37 °C.

**Figure 3:**
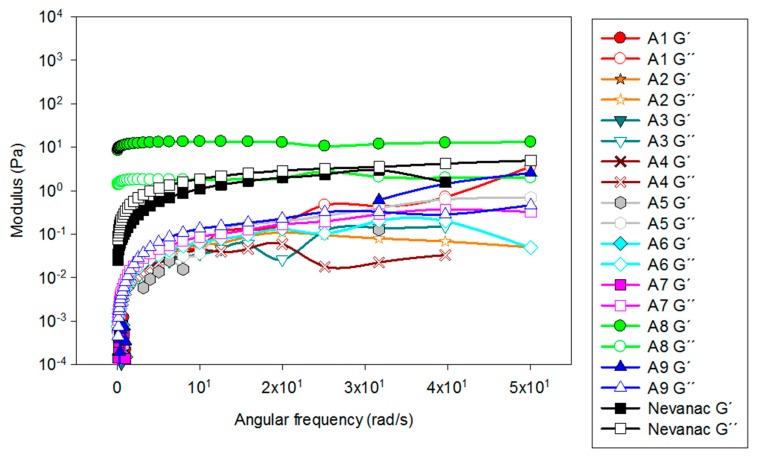
Evolution of storage (G′, solid symbols) and loss (G″, open symbols) moduli as a function of angular frequency (rad/s).

Viscoelastic behavior at 37 °C was also analyzed (Figure 3), recording the dependence of storage (G′) and loss (G″) moduli as a function of angular frequency (rad/s).

Regarding viscoelastic behavior, adding 1% CMC, 0.2% HA, and 0.4% SA to aggregate formulations containing nepafenac/γ-CD/HP-βCD modified the rheological properties of the formulations (Figure 3). Formulations A1 to A7 and A9 performed as very liquid-like systems, and the values of G’ were negligible, showing that they had more viscous than elastic behavior. Alternatively, formulation A8, which contained CMC, HA, and SA, behaved as a well-structured gel (G′ >> G″) and also displayed pseudoplastic behavior. In the case of Nevanac, G′ and G″ values increased with the angular frequency, which is typical of weak gels.

### 3.3. Mucoadhesion Studies

It is known that after topical instillation to the eye, precorneal factors and the reflex mechanism of the eye lead to rapid drug elimination, and only a small fraction of the drug is available at the ocular surface. Treatment of eye diseases with mucoadhesive delivery systems has been proposed as a strategy to enhance the drug retention of topical ophthalmic formulations. Polymer–mucin bonds can be used to trap formulations on the surface of the eye, thus increasing the thickness of the tear film [54]. Several in vitro techniques have been reported to study mucoadhesion [55]. The in vitro tensile test is widely used to assess mucoadhesive strength in terms of the detachment force needed to separate two surfaces [43,56]. The mucoadhesion strength of the formulations is summarized in Table 5.

Nevanac displayed the highest mucoadhesive strength (0.672 ± 0.03N), followed by formulation A4, which contained CMC and HA; A2, which contained CMC; and A5, with PVP, HPMC, and CMC.

Cellulose derivatives such as CMC and HPMC and sodium hyaluronate have been extensively used as mucoadhesive polymers. Brako and co-workers [57] studied the mucoadhesion of progesterone-loaded nanofibers, and found that the addition of CMC to the fibers also increased their mucoadhesion in both artificial and mucosal membranes. Lee et al. [58] found equivalent efficacy in patients with dry eye syndrome treated with HA or CMC eye drops. Mayol et al. [59] designed poloxamer/hyaluronic acid in situ forming hydrogel for drug delivery, showing good mucoadhesion behavior with sustained drug release.

As mucoadhesion is correlated with viscosity [60], these results agree with the viscosity values shown previously. It was reported that the force needed during eye blinking was 0.8 N [50].

### 3.4. Ocular Irritancy Test (HET-CAM)

**Figure 4:**
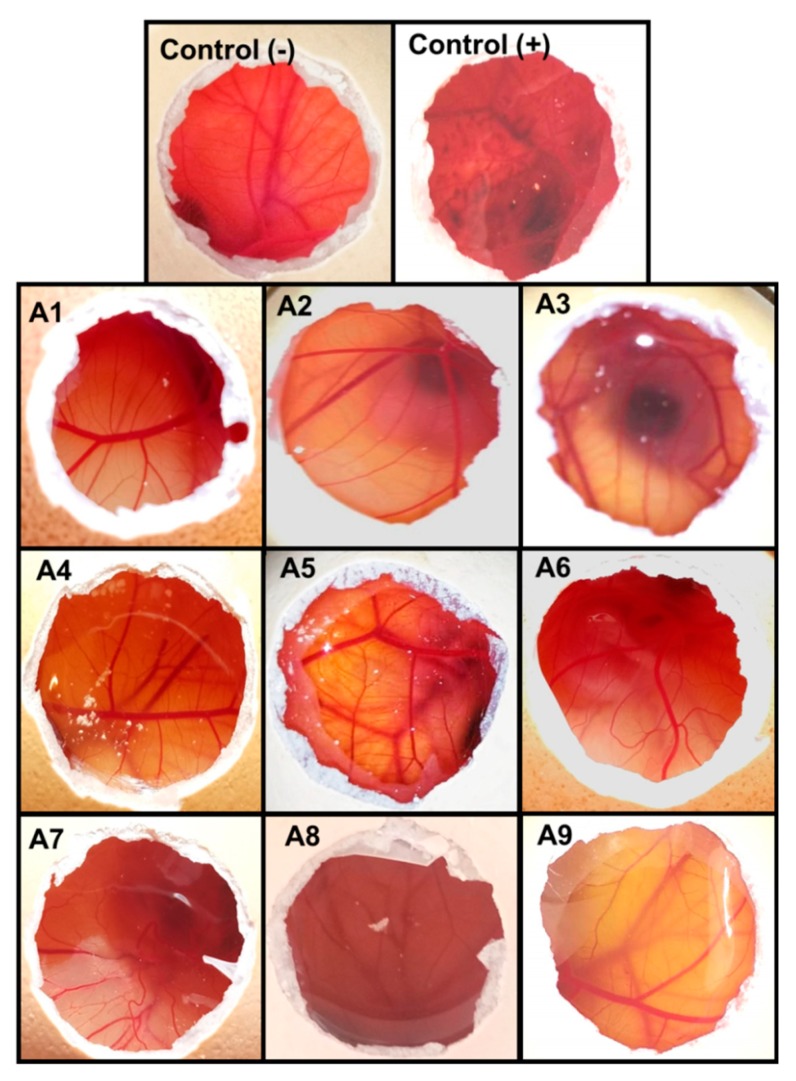
Pictures of Hen’s Egg Test-Chorioallantoic Membrane (HET-CAM) test recorded after five minutes of contact with nepafenac formulations. Negative and positive controls refer to 0.9% NaCl and 0.1 N NaOH, respectively.

The Hen’s Egg Test-Chorioallantoic Membrane (HET-CAM) test is based on the detection of vascular damage in the chorioallantoic membrane, which is an analog for ocular conjunctiva [61]. Different alternatives to the Draize rabbit eye test have been proposed to elucidate the toxicity of potential eye irritants [62,63]. HET-CAM has been described as one of the most suitable alternatives to test eye irritation in vitro since it was found to have good correlation with the Draize test [64]. The HET-CAM assay confirmed that the formulations were not irritants as negative controls (IS = 0) (Figure 4); the IS for the positive control was around 17.

### 3.5. Cell Viability

**Figure 5:**
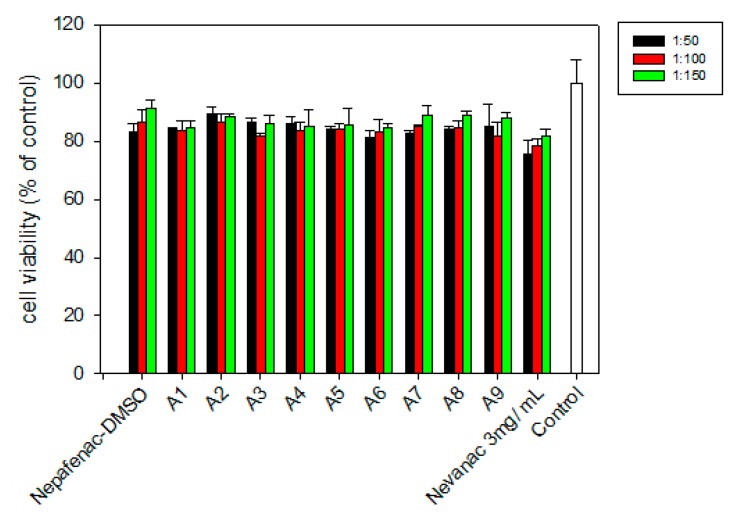
Viability of BALB/3T3 cells after 24 hours of exposure to ophthalmic formulations A1–A9 and Nevanac at various concentrations and control.

The percentage of cell survival relative to the negative control of fibroblasts treated with formulations A1 to A9 and Nevanac at three dilutions is shown in Figure 5.

Nevanac was diluted with DMEM/F12 medium to obtain final concentrations of nepafenac of 0.236 mM, 0.118 mM, and 0.0787 mM, since EC50 was reported to be 0.0875 mM [45]. All formulations were also diluted with DMEM/F12 medium according to these concentrations.

All samples tested were shown to not be harmful to BALB 3T3 cells, with cell viability similar to that exhibited by the marketed formulation, Nevanac. Results confirmed that a dilution of 1:100 was adequate for further assessment of anti-inflammatory activity.

### 3.6. In Vitro Diffusion Studies

**Figure 6:**
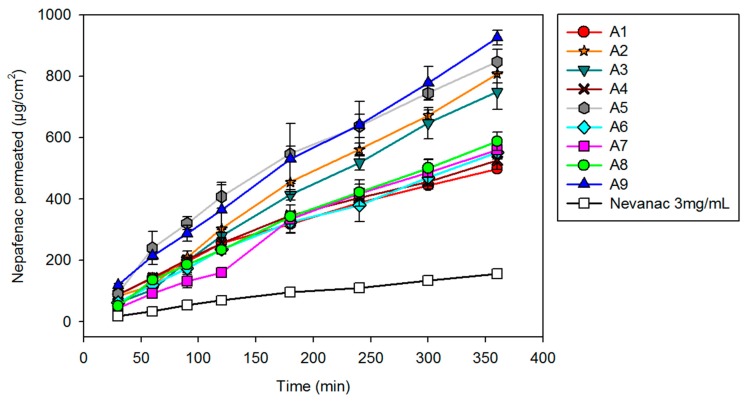
Nepafenac diffusion test through cellulose acetate membrane at 37 °C from eye drop formulations A1 to A9.

Nepafenac diffusion from aggregate formulations and marketed suspension was first evaluated in vitro under sink conditions for six hours. A cellulose acetate membrane (0.45 µm pore size, 25 mm diameter) was used to separate the donor from the receptor compartments (Figure 6).

Formulation A9 showed the fastest diffusion (926.1 ± 23.4 µg/cm^2^), followed by formulation A5 (847.8 ± 39.5 µg/cm^2^), A2 (808.9 ± 31.6 µg/cm^2^), and A3 (749.6 ± 58.4 µg/cm^2^). The increased diffusion compared to Nevanac is due to a higher fraction of solubilized nepafenac. Since diffusion depends on concentration gradient and formulations A2, A3, A5, and A9 are the ones that showed the greatest solubilizing capacity of the drug.

### 3.7. Ex Vivo Corneal and Scleral Permeability Studies

**Figure 7:**
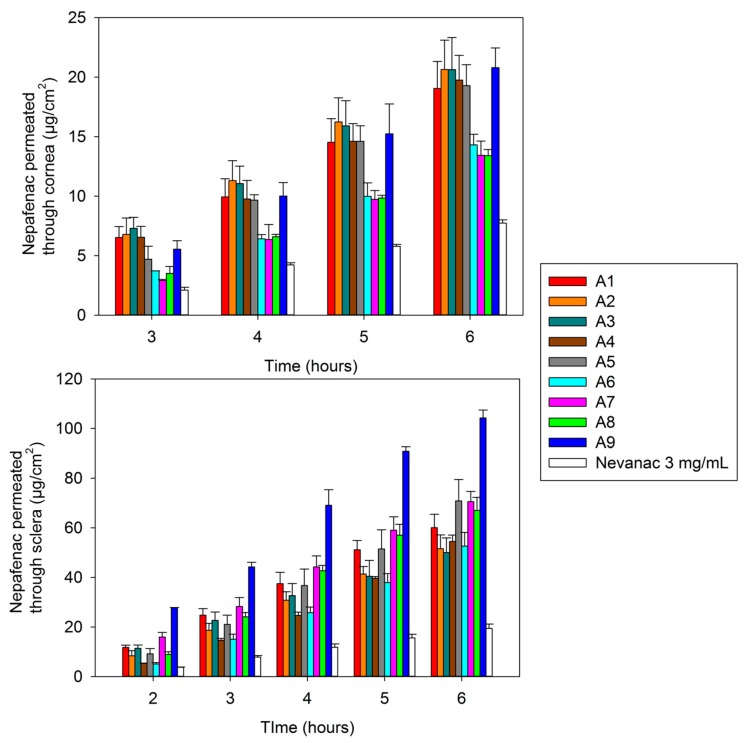
Amount of nepafenac permeated through bovine cornea (**top**) and sclera (**bottom**) measured in the receptor chamber as a function of time.

The amount of permeated nepafenac from selected formulations through bovine cornea and sclera over six hours is shown in Figure 7.

The transcorneal and transscleral permeation profile of nepafenac aggregate formulations was compared with that of the marketed formulation. Nevanac showed the lowest amount of nepafenac permeated through bovine cornea and sclera after six hours compared to nepafenac suspensions, 7.75 ± 0.26 µg/cm^2^ in cornea and 19.44 ± 1.74 µg/cm^2^ in sclera (Figure 7). These findings are in line with those reported in the literature and they could be attributed to the low amount of nepafenac that is solubilized in Nevanac 3 mg/mL (37.87 µg/mL) compared to nepafenac suspension. In fact, various studies using Nevanac and nepafenac-loaded lipid nanoparticles [65] or in situ gels [51] suggested that the low release rate of Nevanac was because it contains Carbopol 974P, which is a highly cross-linked bioadhesive polymer that enables near zero or anomalous release rate.

**Figure 8:**
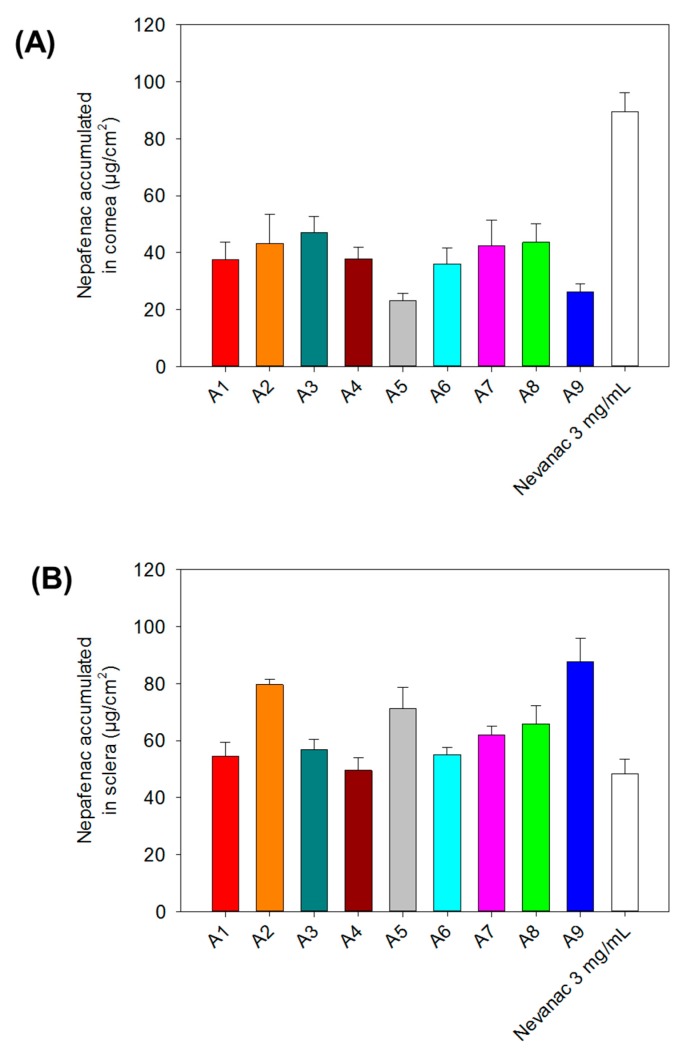
Nepafenac accumulated on the surface and inside (**A**) cornea and (**B**) sclera after six hours of exposure.

All formulations tested showed higher permeability rate compared with Nevanac. This could be attributed to the presence of cyclodextrins in our formulations and a higher fraction of solubilized nepafenac. Numerous studies have shown that CDs enhance drug penetration through biological barriers consisting of an aqueous exterior and a mucosal membrane. Aktaş and co-workers [66] reported that eye drops containing pilocarpine/HPβ-CD complexes demonstrated a four-fold increase in transcorneal penetration compared to a drug formulation without CD. This behavior was also observed by Shelley et al. [51], when they studied the permeability of nepafenac across porcine cornea compared to cyclodextrin formulations of nepafenac. The highest permeability through both bovine cornea and sclera was achieved by formulation A9, 20.80 ± 1.66 µg/cm^2^ in cornea and 104.24 ± 3.21 µg/cm^2^ in sclera, respectively. The amount of nepafenac accumulated at bovine corneal and scleral surfaces and in the tissue after six hours is displayed in Figure 8.

After six hours, Nevanac accumulated the most onto the surface and inside the bovine cornea (89.57 ± 6.66 µg/cm^2^), which can be attributed to the high viscosity of the formulation at the ocular surface. On the other hand, in sclera higher accumulation was found for formulations A9 (87.76 ± 8.08 µg/cm^2^), A8 (65.84 ± 6.34 µg/cm^2^), A5 (71.07 ± 7.64 µg/cm^2^), and A2 (79.64 ± 1.90 µg/cm^2^). These differences between corneal and scleral accumulation are probably due to aggregate formation. Furthermore, consistent with some reports, sclera demonstrated higher permeability compared to cornea [67]. During the recent years, the efficacy of drug delivery to the posterior segment of the eye after topical application of aqueous cyclodextrin-based eye drops has been demonstrated. Loftsson and Stefansson [34] developed eye drops containing dexamethasone/γ-CD complexes to deliver dexamethasone to the posterior segment of the eye to treat diabetic macular edema (DME), which was tested in vivo in rabbits and clinically in patients. They found that the results in DME patients treated with CD eye drops were clinically similar to those after intravitreal corticosteroid injection.

In summary, results of ex vivo sclera accumulation confirmed the potential of our formulations to deliver nepafenac to the posterior segment of the eye via the scleral route.

### 3.8. Anti-Inflammatory Activity

**Figure 9:**
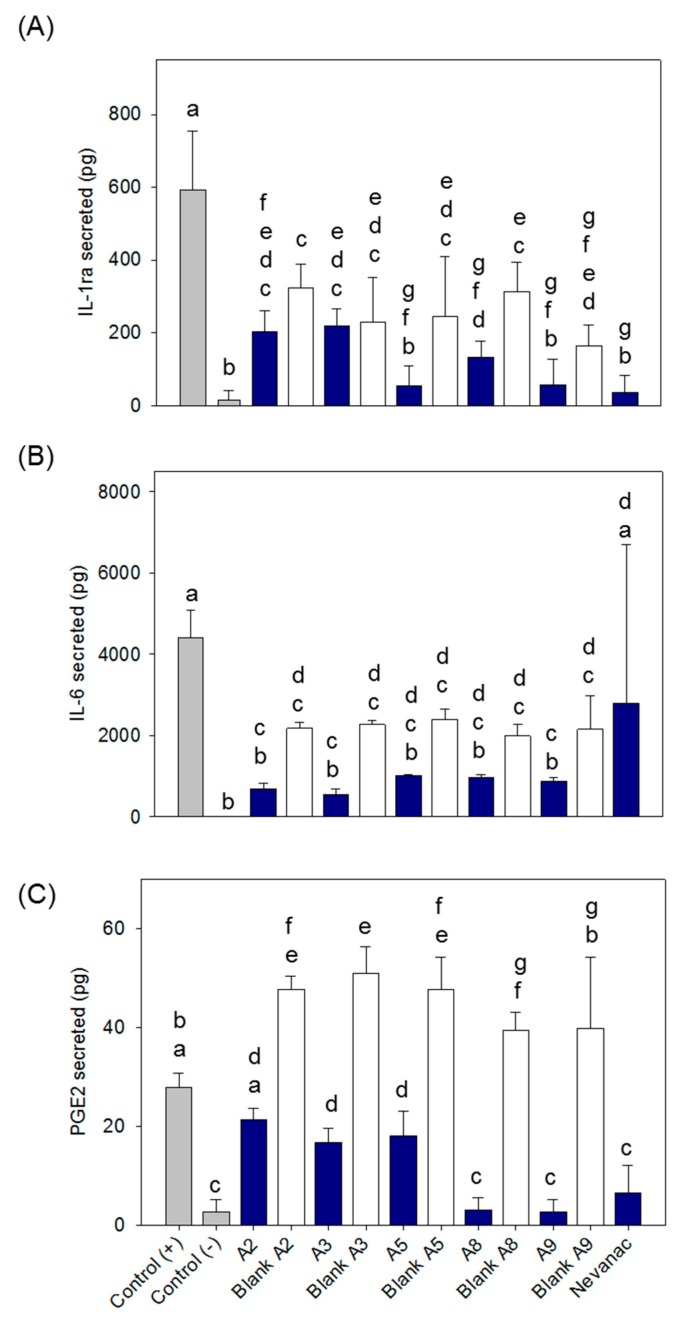
Effect of ophthalmic formulations on the secretion levels of (**A**) interleukin-1 receptor agonist (IL-1ra), (**B**) IL-6, and (**C**) prostaglandin E2 (PGE2) in macrophages. Negative controls refer to unstimulated cells (without lipopolysaccharide (LPS)); positive control refers to cells only stimulated with LPS. Same letters denote statistically homogeneous groups (ANOVA and multiple range test *p* < 0.05; n = 3).

Cytokines and other mediators, such as prostaglandins, play important roles in eye inflammation. Anti-inflammatory drugs used for the treatment of dry eye have been reported to upregulate the production of interleukin 1 (IL-1) receptor antagonist (IL-1ra), among other anti-inflammatory molecules, at the ocular surface [68]. Alternatively, increased levels of IL-6 and prostaglandin E2 (PEG2) have been reported to be involved in dry eye, glaucoma, corneal pathologies, retinal angiogenesis, and diabetic retinopathy progression [69,70]. In this study, we examined the secretion of two pro-inflammatory mediators, IL-6 and PGE2, and one anti-inflammatory mediator, IL-1ra, by macrophages subjected to lipopolysaccharide (LPS) stimulation and treated with the selected formulations to determinate their anti-inflammatory activity (Figure 9).

Figure 9A compares nepafenac loaded formulations to their corresponding blank systems. No significant effect on the secretion of IL-1ra was observed for the developed formulations. The incorporation of nepafenac did not stimulate secretion of this anti-inflammatory molecule. However, a significant reduction in IL-1ra secretion (b-g, ANOVA and multiple range test *p* < 0.05; n = 3) was observed for all formulations compared to the positive control. In the case of IL-6 (Figure 9B), all formulations caused a significant decrease in IL-6 secretion compared to the positive control, reaching levels similar to negative controls (nonstimulated cells) in the case of nepafenac loaded formulations. However, this effect was not observed on Nevanac treated cells. In the case of PGE2 secretion (Figure 9C), Nevanac and nepafenac loaded formulations A3, A5, A8, and A9 significantly reduced the secretion levels of PGE2 compared to the positive controls. Interestingly, A8, A9, and Nevanac treated cells reached levels similar to the negative controls. On the other hand, treatment with loaded A2 and A3 significantly decreased secretion of PGE2 compared to their corresponding blank formulations. In summary, formulations tested A2, A3, A5, A8 and A9 showed a clear in vitro anti-inflammatory effect, reducing the secretion levels of pro-inflammatory molecules (IL-6 and PEG2), without modifying the secretion of anti-inflammatory markers, IL-1ra. In the case of Nevanac, only a reduction in the secretion of PEG2 was observed, which was similar to those detected in formulations A8 and A9. Moreover, formulations A8 and A9 showed the best performance, reaching IL-6 and PGE2 levels similar to non-LPS stimulated cells and superior anti-inflammatory capacity than the commercially available formulation.

Several studies have measured the concentration of inflammatory mediators after treatment with anti-inflammatory drugs to assess their therapeutic activity. Kern et al. [71] analyzed the effect of nepafenac eye drops (0.3%) on PEG2 production in the retina at an early stage of diabetic retinopathy. They found that treatment with nepafenac led to a significant inhibition of PGE2 secretion in the retina. Calles et al. [72] studied the in vitro therapeutic efficacy of dexamethasone-loaded films by measuring changes in IL-6 levels after film exposure using an in vitro model of corneal inflammation. They found that inflamed cells exposed to the dexamethasone films had significantly reduced IL-6 production compared to the controls. In agreement with previously published results, our study points out the effectiveness of formulations loaded with nepafenac, a COX inhibitor, to decrease the secretion of PGE2, improving the performance compared to the commercially available formulation Nevanac. Formulations A8 and A9, containing CDs, CMC, PVA, and MC, showed the most promising data as anti-inflammatory systems.

## 4. Conclusions

In summary, we successfully developed cyclodextrin-based aggregate formulations capable of delivering nepafenac to the posterior segment of the eye via the sclera to treat inflammation. All suspensions were found to be nonirritating and biocompatible after HET-CAM assay and in vitro cell viability assay in murine fibroblasts. The optimal eye drop formulation, A9, containing CMC, PVA, and MC, showed high drug solubilizing capacity, high sclera retention, and a higher reduction of the inflammatory response compared to a marketed formulation, Nevanac^®^ 3 mg/mL. This provides an alternative for the topical delivery of hydrophobic drugs such as nepafenac used to treat ocular diseases at the back of the eye. In the meantime, further studies should be conducted to assess its feasibility in vivo.

## Figures and Tables

**Table 1:** Preliminary nepafenac aggregate formulations. All contain a mixture of 15% (w/v) γ-CD and 8% (w/v) HPβ-CD in aqueous solution [41].

Formulations
F2 = 1.0% (w/v) CMC
F3 = 2.0% (w/v) PVA + 1.0% (w/v) CMC+ 0.1% (w/v) Tyloxapol
F5 = 1.0% (w/v) PVP + 1.0% (w/v) CMC + 0.1% (w/v) HPMC
F9 = 2.0% (w/v) PVA + 1.0% (w/v) CMC + 0.1% (w/v) MC

CD, cyclodextrin; HPβ, hydroxypropyl-β; CMC, carboxymethylcellulose; HPMC, hydroxypropylmethylcellulose; MC, methyl cellulose; PVA, hydrolyzed poly(vinyl alcohol); PVP, polyvinylpyrrolidone.

**Table 2:** Polymers used to prepare nepafenac eye drop formulations and their percentages.

	Eye Drop Formulations								
Component (% w/v)	A1	A2	A3	A4	A5	A6	A7	A8	A9
PVP	–	–	–	–	1.0	–	–	–	–
PVA	–	–	2.0	–	–	–	–	–	2.0
CMC	–	1.0	1.0	1.0	1.0	1.0	–	1.0	1.0
HPMC	–	–	–	–	0.1	–	–	–	–
MC	–	–	–	–	–	–	–	–	0.1
Tyloxapol	–	–	0.1	–	–	–	–	–	–
HA	0.2	–	–	0.2	–	–	–	0.2	–
SA	–	–	–	–	–	0.4	0.4	0.4	–

CMC, carboxymethylcellulose; HPMC, hydroxypropylmethylcellulose; MC, methyl cellulose; PVA, poly(vinyl alcohol); PVP, polyvinylpyrrolidone; HA, sodium hyaluronate; SA, sodium alginate.

**Table 3:** Apparent drug solubility, zeta potential, and pH of nepafenac eye drop suspensions.

Formulation	Apparent Drug Solubility at 25 °C (mg/mL)	Dissolved Drug Content (%)	Zeta Potential (mV)	pH
A1	1.87 ± 0.03	62.33	−10.9 ± 0.6	6.08 ± 0.23
A2	1.95 ± 0.02	65.00	−10.4 ± 0.3	6.18 ± 0.05
A3	2.23 ± 0.01	74.33	−6.4 ± 0.8	6.09 ± 0.03
A4	1.91 ± 0.02	63.66	−12.1 ± 1.4	6.21 ± 0.09
A5	2.50 ± 0.02	83.33	−7.8 ± 0.9	6.07 ± 0.02
A6	1.89 ± 0.05	63.00	−27.4 ± 1.7	6.13 ± 0.33
A7	1.68 ± 0.01	56.00	−14.4 ± 1.4	6.01 ± 0.23
A8	1.95 ± 0.02	65.00	−14.7 ± 1.2	6.16 ± 0.15
A9	2.61 ± 0.02	87.00	−6.9 ± 1.4	6.08 ± 0.05

**Table 4:** Particle size results of diluted aqueous nepafenac eye drops. Data reported are means of three determinations.

	Peak Summary	
Formulation	Size (d. nm)	Intensity (%)
A1	5880.0	73.3
	2619.0	19.9
A2	5880.0	46.9
	4300.0	46.8
	1953.0	6.3
A3	5590.0	96.3
	827.0	3.7
A4	5870.0	100.0
A5	3090.0	53.5
	5950.0	42.0
	481.0	4.5
A6	5575.0	100.0
A7	3380.0	65.1
	5560.0	34.9
A8	4510.0	66.4
	1572.0	33.6
A9	5510.0	77.1
	3250.0	15.3
	340.0	7.6

**Table 5:** Mucoadhesive strength of ophthalmic formulations on ex vivo bovine cornea.

Formulation	Mucoadhesive Strength (N)
A1	0.39 ± 0.15
A2	0.54 ± 0.13
A3	0.41 ± 0.04
A4	0.56 ± 0.11
A5	0.52 ± 0.02
A6	0.39 ± 0.06
A7	0.36 ± 0.08
A8	0.47 ± 0.02
A9	0.38 ± 0.06
Nevanac 3 mg/mL	0.67 ± 0.03
